# In memoriam of Professor Hagop S. Akiskal

**DOI:** 10.1186/s12991-021-00338-2

**Published:** 2021-02-21

**Authors:** Zoltan Rihmer

**Affiliations:** 1 Department of Psychiatry and Psychotherapy, Semmelweis University, Budapest, Hungary; 2 Nyírő Gyula National Institute of Psychiatry and Addictions, Budapest, Hungary

On 20 January, 2021, one of the greatest persons in our profession, Hagop Souren Akiskal, an Armenian–American psychiatrist, have passed away at the age of 77. Dr. Akiskal obtained his medical degree from the American University of Beirut in 1969, and thereafter he settled in the United States and obtained his psychiatric training at the Universities of Tennessee, Memphis, and Wisconsin, Madison. He was appointed Professor of Psychiatry and Pharmacology at the University of Tennessee (1972–1990), and subsequently recruited as the Senior Science Advisor to the Director of the National Institute of Mental Health, Bethesda, Maryland (1990–1994). After this, he became Professor of Psychiatry and Director of the International Mood Center at the University of California at San Diego. However, his scientific and humanistic activity is much more than what could be summarized by these sterile sentences.

One of the deepest professional and personal impressions and influences in my life has been meeting Hagop Akiskal in Budapest during the summer of 1986. That time I have had already read his two, pioneering scientific publications in the field of mood disorders published in the American Journal of Psychiatry in 1977 and in the Archives of General Psychiatry, in 1978. In these fundamental works on the nosological position of cyclothymic disorder and “neurotic depression”, Professor Akiskal demonstrated that cyclothymia and chronic mild depressions were the subaffective/subtle or “embryonal” manifestations of full-blown bipolar and unipolar major mood disorders. These were the first empirical results demonstrating that mild psychopathology did not automatically mean “neurosis” or personality disorder with a psychosocial origin that requires psychotherapy. Based on these results, Professor Akiskal was among the very first to describe the bipolar and unipolar mood spectrum that represents a genetic, biological and pharmacological continuity from mild to severe clinical manifestations of unipolar and bipolar mood disorders. Future independent research, including the findings of my research group, repeatedly replicated and supported these findings. Our scientific and personal contact became close immediately at our first meeting in 1986 when Professor Akiskal have learnt about our findings on the relationship between cyclothymia, polyglottism and bipolar disorder, as well as our studies on the clinical and biological heterogeneity of mild, chronic depression; all of which were in line with his prior findings. “You are my chap…” he said.

In the early 1980s, at the University of Tennessee, he established mood clinics which achieved worldwide appeal because of his philosophy of conducting clinical training and research while delivering high-quality outpatient care. Based on his observations about bipolar patients on long-term lithium therapy showing markedly reduced suicide mortality, he was among the very first to clearly suggest that the key element in suicide prevention was to treat the underlying psychiatric illness. The subsequent works of Professor Akiskal, who was a Distinguished and Emeritus Professor of Psychiatry at the UCSD, focused on depressive mixed states. These works stimulated several American and foreign (Italian, French, German, Hungarian, Norwegian, Portuguese, Greek, etc.) colleagues, whose findings showed a great overlap between depressive mixed states and agitation, and that agitated unipolar depression was the softest cross-sectional clinical expression of bipolarity. This was a rather important step in contemporary nosology, providing new insight into the unipolar–bipolar classification of mood disorders, and showing that there is a continuum between these two, traditionally sharply distinguished, clinical forms.

During the last 30 years, Professor Akiskal’s work on affective temperament has proved to be particularly fruitful for current research. His Temperament Evaluation of the Memphis, Pisa, Paris and San Diego—Autoquestionnaire, shortly called as TEMPS-A scale, has been translated into, and validated in, more than 15 languages. The findings of his studies clearly show the importance of affective temperaments in the development, symptom formation, course, and treatment response of mood disorders. Professor Akiskal’s extensive works on artistic and social creativity widen the everyday clinician’s horizon from the individual to the community level and represent an attractive bridge between biological psychiatry and social psychology. These works, often presented to the general public too, serve as the best way of improving the prestige of psychiatry and destigmatizing psychiatric patients (at least in the field of affective and anxiety disorders), demonstrating that these patients are neither mentally retarded nor “mad”. His literacy on literature, music and fine arts was outstanding and his psychologist wife, Kareen, has been a worthy partner in his research in temperament and creativity.

He was also among the first scientists who raised the possibility that antidepressant monotherapy (unprotected by mood stabilizers or atypical antipsychotics) could induce not only rapid cycling, but could also lead to treatment resistance and might ultimately worsen the cross-sectional clinical picture of depression and this way indirectly induce suicidal behavior. This suggests that the rare cases of antidepressant-induced suicidality may be related to unrecognized bipolarity and mood stabilizer co-therapy could protect the patients from this iatrogeny.

Professor Akiskal had very extensive and intensive national and international research collaborators, including foreign colleagues from Italy, France, Germany, Hungary, Lebanon, Portugal, Norway, Portugal, Spain, Japan, Turkey, Argentina, Brazil, Greece and so forth, and I am happy and proud to share more than 25 scientific publications with him. He have also organized several international symposia and congresses, including the series of International Review of Bipolar Disorders, where, among the top international experts, I have also had the pleasure to be an invited speaker. He also served as invited speaker and presented several key-note lectures at different national and international psychiatric meetings all over the world, including the three International Symposia on Bipolar Disorders, organized in Budapest.

He was an excellent teacher both at undergraduate and postgraduate levels, having won several times the title “Teacher of the Year” at the UCSD. His scientific and presentation capacity, always colored with his own special charm and humor, was also extremely good and well recognized by several national and international psychiatric organizations. As the former Co-Editor-in-Chief of the *Journal of Affective Disorders* with Professor Cornelius Katona, and as a member of the editorial boards of several other premier US and international scientific journals, Professor Akiskal did an amazing amount of work to produce and stimulate new scientific findings and new treatment possibilities in the field of mood and anxiety disorders. He has been honored by all the most prestigious awards in the field of psychiatry. Due to his pioneering works and wide collaborative activity, Professor Akiskal has received several prizes and awards, including the Gold Medal for Pioneer Research (Society of Biological Psychiatry), the German Anna Monika Prize for Depression, the NARSAD Prize for Affective Disorders, the 2002 Jean Delay Prize for international collaborative research (World Psychiatric Association), as well as the French Jules Baillarger and the Italian Aretaeus Prizes for his research in the field of mood disorders including affective temperaments and different clinical manifestations of the unipolar and bipolar mood disorder spectrums. Several dozen studies from different parts of the world have supported and extended his original findings.

There is no doubt that in the last few decades, Professor Akiskal was among the very few top leading psychiatrists in the world. He frequently noted: “Psychiatry is an art, based on scientific methodology”. And really; he has been both a brilliant artist and an excellent methodologist, but first of all a very good clinician as he sometimes described himself a “bipolarist”. His clinical and research activity, mentioned above, revolutionized clinical psychiatry in the last 30–35 years and I am sure he left an indelible print in the history of psychiatry.

